# Clinical Outcomes, Costs, and Value of Surgery Among Older Patients with Colon Cancer at US News and World Report Ranked Versus Unranked Hospitals

**DOI:** 10.1245/s10434-024-16217-5

**Published:** 2024-09-14

**Authors:** Abdullah Altaf, Selamawit Woldesenbet, Muhammad Musaab Munir, Muhammad Muntazir Mehdi Khan, Mujtaba Khalil, Zayed Rashid, Emily Huang, Matthew Kalady, Timothy M. Pawlik

**Affiliations:** 1 Department of Surgery, Division of Surgical Oncology, Health Services Management and Policy, The Urban Meyer III and Shelley Meyer Chair for Cancer Research, The Ohio State University Wexner Medical Center and James Comprehensive Cancer Center, Columbus, OH USA; 2 Department of Surgery, Division of Colorectal Surgery, The Ohio State University Wexner Medical Center and James Comprehensive Cancer Center, Columbus, OH USA

**Keywords:** US News and World Report (USNWR), Colon cancer, Clinical outcomes, Cost, Incremental cost-effectiveness ratio

## Abstract

**Background:**

US News and World Report (USNWR) hospital rankings influence patient choice of hospital, but their association with surgical outcomes remains ill-defined. We sought to characterize clinical outcomes and costs of surgery for colon cancer among USNWR top ranked and unranked hospitals.

**Methods:**

Using Medicare Standard Analytic Files, patients aged ≥65 years undergoing surgery for colon cancer were identified. Hospitals were categorized as ‘ranked’ or ‘unranked’ based on USNWR cancer hospital rankings. One-to-one matching was performed between patients treated at ranked and unranked hospitals, and clinical outcomes and costs of surgery were compared.

**Results:**

Among 50 ranked and 2522 unranked hospitals, 13,650 patient pairs were compared. Overall, 30-day mortality was 2.13% in ranked hospitals versus 3.68% in unranked hospitals (*p* < 0.0001), and the overall paired cost difference was $8159 (*p* < 0.0001). As patient risk increased, 30-day mortality differences became larger, with the ranked hospitals having 30-day mortality of 7.59% versus 11.84% for unranked hospitals among the highest-risk patients (*p* < 0.0001). Overall paired cost differences also increased with increasing patient risk, with cost of care being $72,229 for ranked hospitals versus $56,512 for unranked hospitals among the highest-risk patients (difference = $14,394; *p* = 0.02). The difference in cost per 1% reduction in 30-day mortality was $9009 (95% confidence interval [CI] $6422–$11,597) for lowest-risk patients, which dropped to $3387 (95% CI $2656–$4119) for highest-risk patients (*p* < 0.0001).

**Conclusion:**

Treatment at USNWR-ranked hospitals, particularly for higher-risk patients, was associated with better outcomes but higher-cost care. The benefit of being treated at highly ranked USNWR hospitals was most pronounced among high-risk patients.

**Supplementary Information:**

The online version contains supplementary material available at 10.1245/s10434-024-16217-5.

Colon cancer is the second most prevalent cancer and the second most common cause of cancer-related mortality in the United States.^1^ Additionally, colon cancer has the second highest treatment cost among all cancers, approximately equivalent to $24.3 billion per annum, underscoring its profound burden on the healthcare system.^2^ For many patients with colon cancer, undergoing complex surgery offers their best chance of cure.^3,4^ Previous research has revealed significant variation in the safety of complex surgical procedures, including procedures for colon cancer, across different hospitals. Several quality metrics such as hospital volume, academic affiliation, surgeon experience, specialized facilities, and nurse-to-bed ratio have been associated with variation in clinical outcomes across hospitals.^5–9^ Therefore, for individuals with colon cancer, selecting an appropriate hospital markedly enhances the likelihood of receiving optimal surgical care. However, navigating this decision is complicated by the substantial costs of care and the pervasive issue of financial toxicity, as well as ‘narrow’ insurance networks, making affordability a critical factor in treatment and hospital referral decisions.^10,11^

Hospital reputation influences patient choice of treatment facility. In fact, hospital-related factors such as case-specific volume and academic affiliation are major determinants of patient decision making when selecting their care provider.^12,13^ National ranking systems, such as those featured in US News and World Report (USNWR), bolster hospital reputation by highlighting their commitment to safety and quality and help establish the ‘brand’ of a hospital.^14^ These rankings offer a readily available resource that evaluates both objective and subjective metrics, including hospital structure, processes, outcomes, patient safety, and reputation, to compile rankings across various specialties.^15^ Widespread recognition and annual reporting of these rankings have been linked to positive brand recognition, higher referral and patient volumes, and financial gains for hospitals that achieve top rankings.^16^ Although USNWR hospital rankings are widely recognized and valued by the public, and some studies have explored their correlation with surgical outcomes, the relationship between these rankings and the cost of care for complex procedures, such as colon cancer surgery, remains ill-defined.^17^ This gap in knowledge underscores the need to assess clinical performance of ranked hospitals in relation to financial implications. Furthermore, top-ranked hospitals tend to be more expensive and less transparent about pricing compared with unranked hospitals.^18^ In turn, it remains unclear whether these top-ranked hospitals truly deliver better ‘value’ in terms of improving patient outcomes relative to money spent. In the current study, we sought to quantify the differential impact of surgery for colon cancer at USNWR ranked versus unranked hospitals by comparing clinical outcomes, financial costs, and value (the ratio of difference in cost over difference in mortality).

## Materials and Methods

### Data Source, Study Population, and Variables of Interest

Medicare beneficiaries aged ≥ 65 years with newly diagnosed colon cancer who underwent surgery within 1 year of diagnosis between 2014 and 2021 were identified from the 100% Medicare Standard Analytic Files (SAF) claims data obtained by the Centers for Medicare and Medicaid Services (CMS) using the International Classification of Diseases, Ninth and Tenth Revision (ICD-9 and ICD-10) diagnostic (153.0–153.9, C1 8.0–C18.9) and procedure codes (electronic supplementary material [ESM] Table 1). Patients who were younger than 65.5 years of age at admission, patients who used hospice care, and patients who were not continuously enrolled in Medicare Parts A and B or had health maintenance organization (HMO) enrollment in the 6 months prior to surgery, the month of admission, or the month after admission were excluded. The Strengthening the Reporting of Observational Studies in Epidemiology (STROBE) reporting guidelines were followed in the current study.^19^ The Institutional Review Board (IRB) of the Ohio State University approved this study and waived the need for informed patient consent due to the de-identified nature of the data.

The CMS maintains the Medicare SAF, an administrative billing database that provides patient-level data on patient demographics, diagnoses, procedures, and expenditures. Patient characteristics were defined using the index admission claim and data from Inpatient, Outpatient, and Hospice files 6 months prior to index admission. Data on patient age, sex, race/ethnicity (categorized as White, Black, Hispanic, or other [the ‘other’ category included Asian, American Indian, and Alaska Native]), comorbidities, year of surgery, admission type, encounters at skilled nursing facilities (SNFs) and dialysis facilities, and transfer-in from another hospital’s inpatient or emergency department prior to admission to the index hospital were extracted. Type of admission was predefined as elective, urgent, or emergency in the Medicare database. Urgent and emergency admissions were grouped together as ‘urgent’ in the current study to account for hospital admissions for acutely worsening or life-threatening medical conditions. Outcomes of transferred-in patients were assigned to the hospital where the principal procedure was performed. Each patient’s principal procedure was identified using ICD-9/10 procedure codes (ESM Table 1). Data on each patient’s intensive care unit (ICU) utilization, length of stay, discharge status, readmission within 30 days, survival time, mortality, and health care expenditure/costs were also extracted.

Data on hospital characteristics was obtained from the annual American Hospital Association database.^20^ Hospital-level factors included Council of Teaching Hospital and Health Systems (COTH) membership, geographic region, urban-rural location, hospital bed size, and nurse-to-bed ratio. Regions were defined as Northeast (New England, Middle Atlantic), South (South Atlantic, East South Central, West South Central), Midwest (East North Central, West North Central), and West (Mountain, Pacific) in accordance with the US census.^21^ Hospital bed size was obtained using the total bed count and each hospital’s nurse-to-bed ratio was defined as low, medium, and high, similar to previous studies.^22^

### Primary Exposure and Outcomes of Interest

Hospitals were classified as ‘ranked’ or ‘unranked’ depending on whether the institution appeared in the top 50 cancer hospitals in the 2021 USNWR rankings, and these rankings were consistently applied throughout the duration of the current study.^14^ USNWR assigns rankings to the leading 50 hospitals in each specialty, with those hospitals not ranked assigned the designation of unranked hospitals. Outcomes of interest included 30-day all-cause, all-location mortality, readmission or mortality up to 30 days after discharge from index hospitalization, ICU utilization, length of stay, failure-to-rescue (i.e., mortality rate among patients with postoperative complications), financial costs, and value of care.^23^

Hospital financial performance and spending were evaluated by examining the costs associated with 30-day resource utilization and payments for care, as monitored by the CMS. The costs were calculated based on the total amount Medicare paid for the entire duration of the hospital stay during the index surgery as well as co-insurance payments, deductibles, and third-party payments. Costs related to disproportionate share and indirect medical education were not included in this estimate. To ensure the costs reflected differences in regional living expenses, adjustments were made using the wage index. Furthermore, to account for changes in the cost of healthcare over time, the costs were also adjusted for inflation using the Health Care Price Index.^24,25^ Lastly, the value or incremental cost-effectiveness ratio (ICER) of undergoing surgery at a ranked hospital compared with an unranked hospital was also evaluated. This metric measures the balance between the resources used and the results achieved, providing a way to evaluate the efficiency of resource utilization against the outcomes.^26^ Value or ICER was defined as the difference in financial costs between ranked and unranked hospitals divided by their difference in 30-day mortality, and was reported as the increase in cost or payment for every 1% improvement in mortality, but only if the difference in mortality rates between the two hospital groups was statistically significant. If no statistical difference in mortality was observed, the value was deemed as indeterminate and was not reported.

### Statistical Analyses

The patient cohort was randomly divided into two groups: 90% for matching patients undergoing surgery at ranked and unranked hospitals on a one-to-one basis, and 10% for developing baseline prognostic models.^27^ A patient risk score was calculated to estimate the baseline risk of a patient dying within 30 days of admission. This was achieved by developing binary logistic models that predicted the probability of death within 30 days, using the 10% of the sample set aside for risk modeling (ESM Table 2).^28^ Additionally, prognostic models to predict ICU admission, length of stay, and cost were also developed using the same 10% sample (ESM Tables 3–5).^29–31^ A propensity score for admission to a ranked hospital was also developed.^32^ These risk scores were utilized in matching patients along with specific patient covariates as described below.

Patients from ranked hospitals were matched one-to-one with patients from unranked hospitals using both exact matching and the nearest-neighbor/greedy matching techniques in the Matchit package within the R software program.^33^ Exact matching was performed on the principal procedure type and risk of mortality within 30 days.^34^ Subsequently, within exact groups, a nearest-neighbor matching approach with refined balance to control for patient demographics, admission characteristics, and risk factors was performed.^35^ Adhering to strict balance criteria, the study optimally formed closely matched pairs by reducing the Mahalanobis distance between cases and controls.^36^ This approach considered all comorbidities, demographics, admission factors, risk scores, and the likelihood of being admitted to a ranked hospital to ensure precise pairing. It was aimed to achieve standardized differences in covariate means of <0.1 standard deviations (SDs) following the matching process.^37,38^

McNemar test for binary outcomes and m-statistics for continuous outcomes were used to compare outcomes in matched pairs.^39,40^ Outcomes were also compared between ranked and unranked hospitals inside each risk score quintile. Trends across different risk levels were evaluated using the Mantel test for binary outcomes and robust regression for continuous outcomes.^30,31,41^ Graphs to demonstrate outcome differences between ranked and unranked hospitals by risk level were obtained using the LOWESS procedure in R.^42^ The 95% confidence interval (CI) for ICER values for the value was estimated based on Fieller’s method using STATA.^43^ Additional regression analyses were performed for 30-day mortality and mean adjusted cost of care after adjusting for patient clinicodemographic covariates to minimize any residual imbalance and assess the robustness of the results. All statistical analyses were conducted using SAS 9.4 (SAS Institute, Inc., Cary, NC, USA), STATA version 17 (StataCorp LLC, College Station, TX, USA), and R programming software (R Foundation for Statistical Computing, Vienna, Austria). A *p*‐value <0.05 was considered statistically significant.

## Results

### Patient and Hospital Characteristics

A total of 184,327 Medicare beneficiaries who underwent surgery for colon cancer were included. A small proportion (*n* = 13,650, 7.4%) of patients underwent care at a top 50 ranked hospital, while the majority (*n* = 170,677, 92.6%) underwent care at 3573 unranked hospitals. For the matching process, 100% of the patients from all 50 ranked hospitals were matched to 13,650 (8.0%) patients from 2522 (70.6%) unranked hospitals. Table 1 demonstrates the baseline patient demographics, admission characteristics, comorbidities, principal procedures, prognostic model-predicted outcomes, and the propensity score, highlighting the high quality of matching between the two hospital types, as indicated by standardized differences below 0.1 SDs across all variables.

**Table 1:** Balance of the match for cancer surgery patients: the table reports the balance for selected variables before and after 1:1 matching

Variables (percentage unless otherwise specified)	Matched ranked hospital cases	Matched unranked hospital controls	All unranked hospital controls	Std. diff. before matching	*p*-Value before matching	Std. diff. after matching	*p*-Value after matching
	[*n* = 13,650]	[*n* = 13,650]	[*n* = 170,677]				
Number of hospitals	50	2522	3573	–	–	–	–
Patient demographics							
Age, years							
65–70	25.5	25.4	22.3	0.07	< 0.001	0	0.96
71–75	24.6	24.6	23.2	0.03	< 0.001	0	0.98
76–80	17.5	17.5	17.8	−0.01	0.37	0	1
> 80	32.5	32.5	36.7	−0.09	< 0.001	0	0.98
Sex, male	48	47.6	45.9	0.04	< 0.001	0.01	0.58
Race							
White	84.1	84.5	87.2	−0.08	< 0.001	−0.01	0.46
Black	7.1	7	7.6	−0.02	0.06	0	0.76
Hispanic	1.1	1	1.1	0	0.85	0	0.68
Other	7.6	7.5	4.1	0.13	< 0.001	0.01	0.58
Year of treatment							
2014	11.4	11.5	13.4	−0.06	< 0.001	0	0.85
2015	12.2	12.1	13.9	−0.05	< 0.001	0	0.72
2016	13.8	14.4	14	−0.01	0.47	−0.02	0.17
2017	13.3	13.3	13.6	−0.01	0.31	0	0.89
2018	13.8	13.8	13.2	0.02	0.045	0	0.94
2019	14.3	14	12.5	0.05	< 0.001	0.01	0.42
2020	11.5	11.4	10.7	0.02	0.004	0	0.85
2021	9.7	9.5	8.6	0.03	< 0.001	0.01	0.59
Urgent surgery	20.6	20.6	35.6	−0.37	< 0.001	0	0.95
Transfer-in	2	2	1.2	0.1	< 0.001	0	0.9
Skilled nursing facility visit	3.4	3.1	4.2	−0.05	< 0.001	0.02	0.12
Dialysis	0.8	0.8	0.8	0	0.69	0	0.84
Comorbidities							
Congestive heart failure	15.4	15.5	18.2	−0.08	< 0.001	0	0.84
Chronic pulmonary disease	19.3	19.4	22.5	−0.08	< 0.001	0	0.93
Dementia	3.5	3.4	5.5	−0.11	< 0.001	0.01	0.49
Liver disease	6.5	6.3	4.4	0.1	< 0.001	0.01	0.66
Diabetes mellitus	0.9	0.9	1	−0.01	0.18	0	0.9
Renal disease	17.4	17.3	18.1	−0.02	0.041	0	0.92
Rheumatologic disease	3.2	3	2.9	0.02	0.039	0.01	0.38
Stroke	0.9	0.8	1	−0.02	0.046	0	0.95
Principal procedure							
Open colectomy	40.3	40.3	49.1	−0.20	< 0.001	0	1
Laparoscopic colectomy	12.2	12.2	10.9	0.04	< 0.001	0	1
Excision via percutaneous approach	6.7	6.7	5.4	0.05	< 0.001	0	1
Resection via percutaneous approach	28.2	28.2	22.3	0.13	< 0.001	0	1
Excision via natural or artificial opening	9.9	9.9	11	−0.04	< 0.001	0	1
Robotic surgery	2.8	2.8	1.4	0.09	< 0.001	0	1
Predictions based on prognostic models							
Probability of 30-day mortality [% (95% CI)]	0.02 (0.01–0.04)	0.02 (0.01–0.04)	0.03 (0.02–0.06)	−0.32	< 0.001	0	0.98
Predicted cost [$ (95% CI)]	41,800.70	41,800.70	43,269.10	−0.24	< 0.001	0	0.98
	(36,252.3–47,237.1)	(36,235.1–47,223.0)	(38,167.4–50,564.0)				
Probability of ICU utilization [% (95% CI)]	0.21 (0.15–0.33)	0.21 (0.15–0.33)	0.26 (0.17–0.4)	−0.28	< 0.001	0	0.99
Predicted length of stay [days (95% CI)]	4.81 (4.03–6.32)	4.81 (4.03–6.3)	5.35 (4.43–8.53)	−0.39	< 0.001	0	0.99
Propensity score (95% CI)	0.08 (0.06–0.09)	0.08 (0.06–0.09)	0.07 (0.06–0.09)	0.29	< 0.001	0.01	0.67
Hospital characteristics (not included in matching)							
Mean number of cases per hospital	273	–	47.8	–	< 0.001	–	< 0.001
COTH membership	90.3	16.1	16.1	–	< 0.001	–	< 0.001
Total number of beds [median (IQR)]	889 (605–1367)	309 (108–530)	307 (121–530)	–	< 0.001	–	< 0.001
Hospital status				–	< 0.001	–	< 0.001
Rural	62.5	29.4	29.4				
Urban	37.5	70.6	70.6				
Nurse-to-bed ratio				–	< 0.001	–	< 0.001
Low	76	32	32				
Medium	23.2	33.4	33.4				
High	0.8	34.6	34.6				

*Std diff.* standardized difference, *COTH* Council of Teaching Hospitals and Health Systems, *CI* confidence interval, *IQR* interquartile range

Of note, the mean number of cases was 273 per hospital for ranked hospitals and 47.8 per hospital for unranked hospitals (*p* < 0.001). Ranked hospitals were more likely to have COTH membership (90.3% vs. 16.1%) and a greater median number of beds {889 (interquartile range [IQR] 605–1367) vs. 307 (IQR 121–530)} compared with unranked hospitals (both *p* < 0.001). Most ranked hospitals had a low nurse-to-bed ratio (76%), with the nurse-to-bed ratios being more evenly distributed among unranked hospitals.

### Clinical and Financial Outcomes Relative to US News and World Report Ranking

Overall, 30-day mortality was lower at ranked versus unranked hospitals (2.13% vs. 3.68%; difference −1.55%; *p* <  0.0001) (Table 2). Although ICU utilization was similar between the two groups (24.59% in ranked hospitals vs. 25.12% in unranked hospitals; *p* = 0.282), failure-to-rescue rates were much lower at ranked hospitals (6.59% vs. 10.48%; difference −3.89%; *p* = 0.0001). Moreover, ranked hospitals had a lower incidence of 30-day readmission or death (15.46% vs. 16.53%; difference −1.07%; *p* = 0.014) and a slightly reduced average length of stay (3.48 days vs. 3.60 days; *p* = 0.0015). Notably, the mean adjusted cost of care was considerably higher at ranked hospitals versus unranked hospitals ($55,996 vs. $45,279; difference + $8159; *p* <  0.0001).

**Table 2:** Clinical outcomes, costs, and incremental cost-effectiveness ratio by hospital type and patient risk on admission

Outcome (percentage unless otherwise specified)	Cohort	All patients	Quintiles of risk on admission (Q1 = lowest, Q5 = highest)					Trend *p*-value
			Q1	Q2	Q3	Q4	Q5	
Number of pairs		13,650	3792	3108	2996	2081	1673	
30-day mortality	Ranked hospital cases	2.13	0.29	0.87	1.67	3.65	7.59	< 0.0001
	Unranked hospital controls	3.68	0.82	1.93	2.67	6.39	11.84	
	Difference in outcome	−1.55	−0.53	−1.06	−1.00	−2.74	−4.25	
	*P*-value	< 0.0001	0.002	0.0003	0.0075	< 0.0001	< 0.0001	
ICU utilization	Ranked hospital cases	24.59	12.34	18.47	23.77	36.86	49.91	< 0.0001
	Unranked hospital controls	25.12	11.92	18.92	24.87	38.49	50.39	
	Difference in outcome	−0.53	0.42	−0.45	−1.1	−1.63	−0.48	
	*P*-value	0.282	0.569	0.647	0.321	0.270	0.781	
Failure-to-rescue	Ranked hospital cases	6.59	0	3.42	5.13	5.34	9.54	< 0.0001
	Unranked hospital controls	10.48	0	4.79	5.64	10.92	14.47	
	Difference in outcome	−3.89	0	−1.37	−0.51	−5.58	−4.93	
	*P*-value	0.0001	–	0.564	0.819	0.003	0.008	
30-day readmission or death	Ranked hospital cases	15.46	8.83	12.42	14.45	21.77	30.07	< 0.0001
	Unranked hospital controls	16.53	9.39	13.45	15.15	24.46	31.02	
	Difference in outcome	−1.07	−0.56	−1.03	−0.7	−2.69	−0.95	
	*P*-value	0.014	0.410	0.231	0.440	0.037	0.550	
Length of stay, days	Ranked hospital cases	3.48	3.54	4.46	5.25	7.89	10.71	0.894
	Unranked hospital controls	3.60	3.60	4.59	5.21	7.74	10.32	
	Difference in outcome	−0.12	−0.06	−0.13	−0.04	−0.15	0.39	
	*P*-value	0.0015	0.168	0.026	0.610	0.301	0.079	
Mean adjusted cost of care, $	Ranked hospital cases	55,996	41,373	55,165	56,914	69,623	72,229	< 0.0001
	Unranked hospital controls	45,279	36,428	44,092	45,962	57,455	56,512	
	Difference in cost	8159	4775	8350	8963	11,416	14,394	
	*P*-value	< 0.0001	< 0.0001	0.018	0.0002	< 0.0001	0.02	
	Difference in 30-day mortality	−1.55	−0.53	−1.06	−1.00	−2.74	−4.25	
	Value of care/ ICER	$5264	$9009	$7877	$8963	$4166	$3387	
	95% CI	$4642–$5889	$6422–$11,597	$5807–$9976	$6837–$11,122	$3086–$5250	$2656–$4119	

*ICU* intensive care unit, *ICER* incremental cost-effectiveness ratio, *CI* confidence interval

**Fig. 1:**
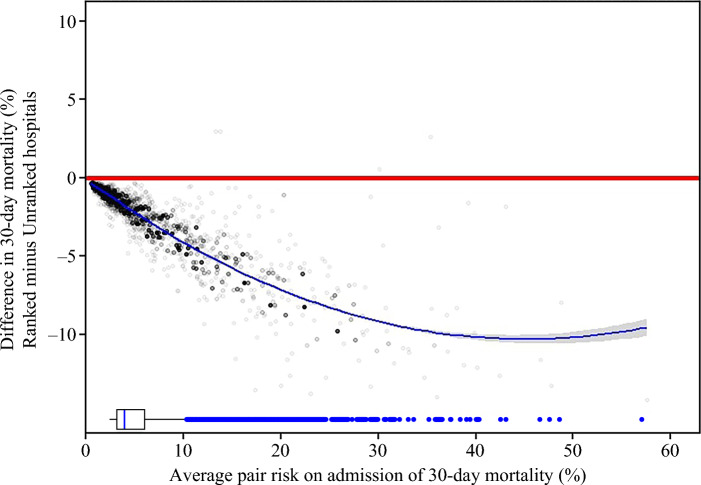
LOWESS plot of difference in mortality rate (%) between matched pairs of patients undergoing surgery for colon cancer at ranked and unranked hospitals versus risk of 30-day mortality on admission

Analysis stratified by quintiles of risk at admission (risk synergy), with Q1 being the lowest-risk quintile and Q5 being the highest-risk quintile, revealed a significant trend of increased differences in outcomes among ranked and unranked hospitals (Table 2). Ranked versus unranked hospitals demonstrated a greater difference in 30-day mortality as patient risk increased from −0.53% in Q1 (0.29% vs. 0.82%; *p* = 0.002) to −4.25% in Q5 (7.59% vs. 11.84%; *p* < 0.0001), indicating a greater survival benefit for higher-risk patients (risk trend *p* < 0.0001) [Fig. 1]. In contrast, ICU utilization among ranked and unranked hospitals demonstrated minimal variance with relatively consistent utilization across all five risk quintiles (all *p* > 0.05).

**Fig. 2:**
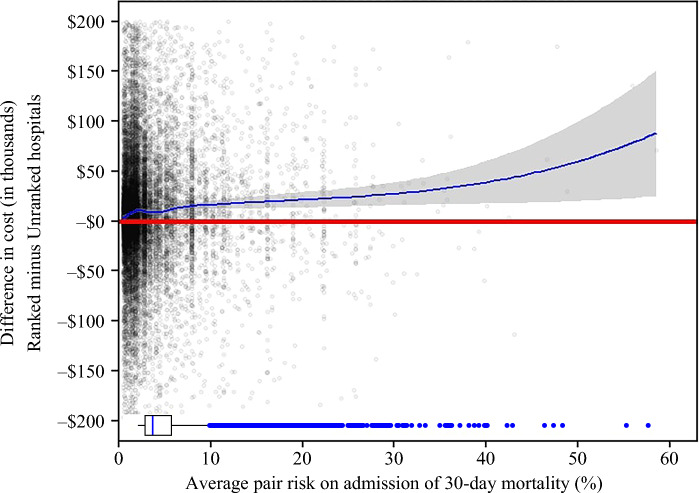
LOWESS plot of difference in cost of care ($) between matched pairs of patients undergoing surgery for colon cancer at ranked and unranked hospitals versus risk of 30-day mortality on admission

Failure-to-rescue rates exhibited greater differences at higher patient risk levels, culminating in a −5.58 difference in Q4 (5.34% vs. 10.92%; *p* = 0.003) and a −4.93% difference in Q5 (9.54% vs. 14.47%; *p* = 0.008), reflecting far better rescue rates in ranked hospitals for patients with the highest risk (risk trend *p* < 0.0001). Although there was no difference in 30-day readmission or risk of death at the lowest risk levels (−0.56% difference; *p* = 0.410), the highest-risk patients who underwent care at ranked hospitals demonstrated a marked reduction in 30-day readmission or death (Q5: 21.77% vs. 24.46%, difference −2.69%; *p* = 0.037) versus unranked hospitals. Length of stay demonstrated negligible differences in the lower quintiles, with length of stay generally being lower in ranked versus unranked hospitals. However, ranked hospitals had higher length of stay (10.71 days vs. 10.32 days, difference 0.39 days; *p* = 0.079) compared with unranked hospitals in the highest-risk quintile. Mean adjusted cost of care rose with patient risk at admission at ranked versus unranked hospitals, starting from a difference of +$4775 in Q1 ($41,373 vs. $36,428; *p* < 0.0001) to +$14,394 in Q5 ($72,229 vs. $56,512; *p* = 0.02) [risk trend *p* < 0.0001] (Fig. 2).

After adjusting for hospital characteristics, including total number of beds, COTH membership status, hospital location (rural/urban), and nurse-to-bed ratio, treatment at unranked hospitals remained associated with higher odds of 30-day all cause, all-location mortality (unranked hospitals vs. ranked hospitals: odds ratio [OR] 1.91, 95% CI 1.90–1.92; *p* < 0.0001). Moreover, an additional regression analysis was conducted post-matching to adjust for patient clinicodemographic covariates to further minimize residual imbalances. This analysis confirmed the initial findings, demonstrating lower odds of 30-day mortality and higher cost of care at ranked hospitals compared with unranked hospitals across matched groups (ESM Table 6).

### Value of Care/Incremental Cost-Effectiveness Ratio

**Fig. 3:**
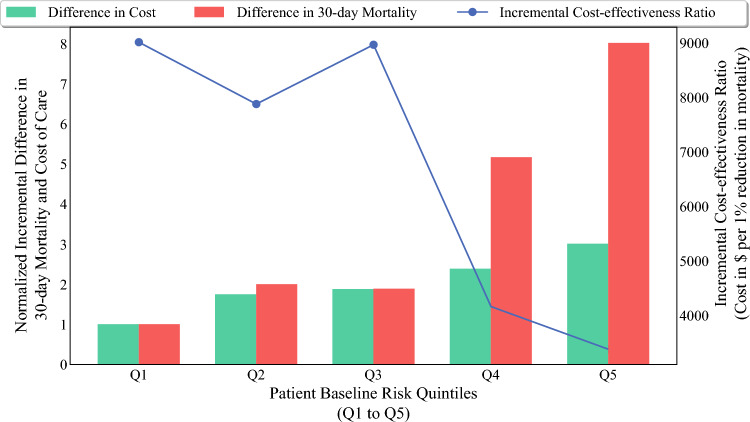
Comparison of normalized differences in 30-day mortality and cost of care across patient risk quintiles (Q1–Q5) and ICER; green bars represent cost differences, red bars represent mortality differences (both normalized to Q1 values), black line shows ICER in US$ per 1% reduction in mortality, with the left y-axis indicating normalized differences and the right y-axis showing ICER. *ICER* incremental cost-effectiveness ratio, *US$* United States dollars

Using mean adjusted cost of care, the value or ICER was $5264 (95% CI $4642–$5889) for a 1% reduction in 30-day mortality among patients with colon cancer undergoing surgery at ranked versus unranked hospitals. As the baseline patient risk at admission increased, the gap in the cost of care between ranked and unranked hospitals expanded; of note, the decrease in 30-day mortality demonstrated a more substantial difference (Fig. 3). As a result, there was a decline in the ICER as admission risk levels increased. Notably, there was an approximately threefold decrease in the ICER from Q1 ($9009, 95% CI $6422–$11,597) to Q5 ($3387, 95% CI $2656–$4119) for a 1% reduction in 30-day mortality (Table 2).

## Discussion

National ranking systems such as the USNWR bolster hospital reputations by emphasizing safety and quality commitment, thereby establishing hospital brands.^12–14^ These rankings evaluate various metrics, including structure, processes, outcomes, safety, and reputation, aiding comparisons across specialties. Such rankings drive positive brand recognition, higher referrals, patient volumes, and quality of care for top-ranked hospitals.^44^ Nonetheless, care at these top-ranked hospitals may also be associated with higher financial expenditures, and their performance and value of care relative to clinical outcomes, especially for patients undergoing complex procedures, is a matter of ongoing debate among providers and payers.^45,46^ Therefore, the current study was important as we characterized variations in clinical outcomes and financial costs among patients undergoing surgery for colon cancer at USNWR ranked versus unranked hospitals. Using matched pairs from a nationally representative dataset, the current work demonstrated that top-ranked hospitals had better overall clinical outcomes compared with unranked hospitals. In addition, there was an increasing benefit of treatment at ranked hospitals among patients with poorer health or higher risk of mortality on admission. However, surgical care at ranked hospitals was associated with a greater financial burden that increased with baseline patient risk. Of note, the value of care or ICER for surgical care demonstrated a positive association with increasing patient risk, indicating a trend in which care became more cost effective at ranked hospitals as patient risk worsened.

Variations in clinical outcomes across specialties are closely tied to the hospital in which care is received. Therefore, the choice of hospital for surgery, particularly for cancer surgery, is a crucial decision that can influence patient outcomes, surgical safety, and the overall success of the treatment.^47,48^ Efforts to improve outcomes have largely focused on improving hospital quality, e.g. through programs such as the Surgical Care Improvement Project by CMS that promotes adherence to evidence-based perioperative care practices through pay-for-performance initiatives.^49^ However, patient-centric metrics such as the USNWR hospital rankings enable individuals to compare hospitals conveniently based on diverse outcomes across numerous specialties.^14^ Annually, USNWR evaluates around 5000 medical centers spanning 25 specialties, including cancer, gastrointestinal surgery, cardiology, and orthopedics. Within each specialty, the top 50 hospitals are ranked, with the top 20 earning prestigious honor-roll status, indicating exceptional surgical care quality.^50^ Despite this perceived influence, there is limited information on quality differences between ranked and unranked hospitals among patients undergoing cancer surgery. Nevertheless, hospital reputation shapes patient decision making regarding choice of hospital for clinical care as rankings influence human psychology, leading patients to associate top USNWR rankings with superior care due to attentional biases and the prestige of high-ranked hospitals evoking positive emotions, which drive decisions based on psychosocial factors rather than specific health needs.^51,52^ In fact, a survey conducted by Ellis et al. revealed that 61.9% of participants considered hospital rankings as the most critical factor in selecting a hospital.^53^ The current work examined the relationship between USNWR rankings and clinical outcomes among a 1:1 balanced cohort of patients on a national scale and demonstrated that receiving surgical care at top-ranked hospitals was associated with improved short-term mortality and morbidity, particularly for the highest-risk patients. These findings are in line with a previous study by Wang et al. that demonstrated improved cardiovascular outcomes among patients undergoing treatment at top-ranked hospitals.^54^ The influence of USNWR rankings on hospital selection most likely impacts choice related to elective surgical cases, as patients requiring urgent care often do not have the opportunity to consider these rankings in their decision-making process.

An improvement in clinical outcomes at ranked hospitals is likely a surrogate for higher annual case volume.^55^ Ranked hospitals are often research-focused academic tertiary care centers with larger patient volumes, integrating the expertise of various medical professionals, including surgeons, anesthesiologists, radiologists, operating room staff, intensivists, and nurses, to offer multidisciplinary, patient-centered care.^56–58^ The current study also demonstrated that ranked hospitals were more likely to have COTH membership (90.3% vs. 16.1%; *p* < 0.001), as well as a markedly higher mean number of cases (273 vs. 47.8; *p* < 0.001) and median number of beds (889 vs. 307; *p* < 0.001). Nonetheless, there was an increase in mean adjusted cost of care among patients receiving care at ranked versus unranked hospitals. The rate of ICU utilization among ranked versus unranked hospitals was largely comparable. The relatively high ICU utilization rate observed in both ranked and unranked hospital groups may have been attributed to the matched case-control design, in which the selection of controls was dependent on matching with cases. In addition, ICU care may have included patients receiving progressive care unit (PCU)/stepdown care, which may have elevated the reported use of these higher-level care units. Of note, ranked hospitals had much lower failure-to-rescue rates, especially for the highest-risk patients (9.54% vs. 14.47%, difference −4.93%; *p* = 0.008). In turn, ‘rescuing’ patients from perioperative complications is costly and resource intensive.^59^ Therefore, mean adjusted surgical costs at ranked hospitals may have been driven, in part, by greater case complexity, higher resource allocation, advanced medical facilities, and elevated staffing levels.^60^ Additionally, longer hospital stays required to manage complex cases may also contribute to higher cost of care. Although our findings indicate comparable lengths of stay across most patient risk groups, the direct contribution of increased length of stay to overall costs merits further investigation. Specifically, further studies should seek to define how extended care at ranked hospitals influences the cost-effectiveness ratio, thereby affecting the overall value proposition versus unranked hospitals.

Surgery represents a significant expenditure for Medicare services, as surgical episodes of care can be expensive, particularly in relation to in-hospital procedures. On 1:1 matching analysis, the ICER for surgery at ranked versus unranked hospitals decreased approximately threefold, from $9009 to $3387, for a 1% reduction in 30-day mortality among patients with the highest baseline risk on admission. These findings suggested that ranked hospitals may offer a more efficient and cost-effective alternative to unranked hospitals in rescuing patients from major complications. These data may assist policymakers and healthcare providers in optimizing patient care and resource allocation, particularly amid resource constraints and increasing demand for healthcare services. Improving patient outcomes, particularly for high-risk individuals, may be feasible in unranked hospitals despite challenges in implementing gross structural changes by strengthening multidisciplinary care models.^61^ Collaborative partnerships with higher-ranked institutions can enhance the sharing of clinical knowledge and resources, ultimately raising the standard of care in unranked hospitals.^62^ Policy interventions are essential to address outcome disparities between ranked and unranked hospitals, especially for high-risk patients. These interventions encompass empowering patient choice and transparency through information about hospital rankings and quality metrics, transitioning to payment models that incentivize value-based care, addressing healthcare disparities by investing in underserved communities, encouraging referrals of high-risk patients to specialized care facilities, and ensuring equitable access to high-quality care for marginalized communities.^63–65^ In tandem with these efforts, addressing the Matthew Effect—an economic principle in which resources tend to be disproportionately allocated to those hospitals already well-resourced, further widening healthcare inequities—is crucial.^66^ This phenomenon contributes to disparities in healthcare access, as top-ranked hospitals continue to attract more investments and recognition, leaving underresourced institutions, often serving the most disadvantaged populations, with fewer opportunities to improve and provide high-quality care.^67^ Through these targeted interventions and strategic healthcare planning, all patients need to be provided with access to the best possible care, advancing toward a more equitable and effective healthcare system.

The current study should be interpreted in light of several limitations. As with any retrospective study, selection bias was possible. Use of a large administrative dataset has inherent limitations given the reliance on diagnosis and procedural codes from billing data. Due to the utilization of Medicare claims data, it was likely that racial/ethnic minorities were somewhat underrepresented. The Medicare database included only patients aged 65 years and older, limiting generalizability to other younger and privately insured patient populations. Additionally, data on out-of-pocket (OOP) spending were not assessed, which limited the ability to directly assess patient financial exposure and the element of financial toxicity. Moreover, the Medicare Prospective Payment System (PPS) may influence variations in hospital spending, with PPS-exempt hospitals receiving higher reimbursements due to management of complex cases that require extensive resources.^68^ Understanding the impact of these exemptions on hospital performance and patient cost burden may be important. The analysis focused on USNWR rankings at a single time point to ensure consistency and facilitate accurate comparisons, avoiding the complexity of fluctuating rankings. Although USNWR accounts for disease severity and complexity in its evaluation of hospitals, due to the nature of the dataset, the current study could not adjust for detailed clinicopathologic factors such as disease stage, histologic characteristics, tumor burden, operative blood loss, and surgical margins. Despite its limitations, Medicare user files represent one of the largest patient population cohorts in the United States.

## Conclusion

In summary, among comparable patients, individuals undergoing colon cancer surgery at ranked hospitals experienced lower 30-day mortality rates versus unranked hospitals. Despite increased financial costs associated with higher patient risk, there were clinical benefits provided by ranked hospitals. Notably, among Medicare patients who underwent surgery for colon cancer, the benefit of treatment at ranked hospitals became more evident as patient admission risk of mortality increased. Overall, cost of care was higher at ranked hospitals but appeared to return good value for the money spent, especially among the highest-risk patients. These data may help inform patient opinion around USNWR and surgical hospital referrals, as well policies related to resource allocation to ensure healthcare equity.

## Supplementary Information

Below is the link to the electronic supplementary material.

Supplementary file1 (DOCX 39 KB)

## Data Availability

The data for this study were obtained from the Medicare SAFs. There are restrictions to the availability of this data, which is used under license for this study. Data can be accessed with permission from the CMS.
