# Supplementary material for: Clinical Outcomes, Costs, and Value of Surgery Among Older Patients with Colon Cancer at US News and World Report Ranked Versus Unranked Hospitals
Source: Ann Surg Oncol. 2024 Sep 14;31(13):8517–29. doi: 10.1245/s10434-024-16217-5 (PMC11549124; doi:10.1245/s10434-024-16217-5)
Supplement: Supplementary file 1 — Supplementary file1 (DOCX 39 KB) [file 10434_2024_16217_MOESM1_ESM.docx]

**Supplementary Table 1.** ICD-9/10 Procedure Codes for Colon Cancer

| Surgery for Colon Cancer | ICD-9 Codes | ICD-10 Codes |
| --- | --- | --- |
| Laparoscopic multiple segmental resections of large intestine | 17.31 | 0DBE4ZZ |
| Laparoscopic cecectomy | 17.32 | 0DTH4ZZ |
| Laparoscopic right hemicolectomy | 17.33 | 0DTF4ZZ |
| Laparoscopic resection of transverse colon | 17.34 | 0DTL4ZZ |
| Laparoscopic left hemicolectomy | 17.35 | 0DTG4ZZ |
| Laparoscopic sigmoidectomy | 17.36 | 0DTN4ZZ |
| Other laparoscopic partial excision of large intestine | 17.39 | 0DBE4ZZ |
| Excision of Right Large Intestine, Percutaneous Endoscopic Approach |  | 0DBF4ZZ, |
| Excision of Left Large Intestine, Percutaneous Endoscopic Approach |  | 0DBG4ZZ, |
| Excision of Cecum, Percutaneous Endoscopic Approach |  | 0DBH4ZZ |
| Excision of Ascending Colon, Percutaneous Endoscopic Approach |  | 0DBK4ZZ |
| Excision of Transverse Colon, Percutaneous Endoscopic Approach |  | 0DBL4ZZ |
| Excision of Descending Colon, Percutaneous Endoscopic Approach |  | 0DBM4ZZ |
| Excision of Sigmoid Colon, Percutaneous Endoscopic Approach |  | 0DBN4ZZ |
| Resection of Ascending Colon, Percutaneous Endoscopic Approach |  | 0DTK4ZZ |
| Resection of Descending Colon, Percutaneous Endoscopic Approach |  | 0DTM4ZZ  0DTEFZZ |
| Resection of Right Large Intestine, Percutaneous Endoscopic Approach |  | 0DTF4ZZ  0DTFFZZ |
| Resection of Left Large Intestine, Percutaneous Endoscopic Approach |  | 0DTG4ZZ |
| Resection of Left Large Intestine, Via Natural or Artificial Opening with Percutaneous Endoscopic Assistance |  | 0DTGFZZ |
| Resection of Left Large Intestine, Open Approach |  | 0DTG0ZZ |
| Resection of Cecum, Percutaneous Endoscopic Approach |  | 0DTH4ZZ  0DTHFZZ |
| Resection of Ascending Colon, Percutaneous Endoscopic Approach |  | 0DTK4ZZ |
| Resection of Ascending Colon, Via Natural or Artificial Opening Endoscopic |  | 0DTK8ZZ  0DTKFZZ |
| Resection of Ascending Colon, Open Approach |  | 0DTK0ZZ |
| Resection of Ascending Colon, Via Natural or Artificial Opening |  | 0DTK7ZZ |
| Resection of Transverse Colon, Percutaneous Endoscopic Approach |  | 0DTL4ZZ |
| Resection of Descending Colon, Percutaneous Endoscopic Approach |  | 0DTM4ZZ |
| Resection of Descending Colon, Via Natural or Artificial Opening Endoscopic |  | 0DTM8ZZ |
| Resection of Descending Colon, Open Approach |  | 0DTM0ZZ |
| Resection of Descending Colon, Via Natural or Artificial Opening |  | 0DTM7ZZ |
| Resection of Sigmoid Colon, Percutaneous Endoscopic Approach |  | 0DTN4ZZ |
| Excision of Large Intestine, Percutaneous Endoscopic Approach |  | 0DBE4ZZ  0DBEFZZ |
| Excision of Right Large Intestine, Percutaneous Endoscopic Approach |  | 0DBF4ZZ |
| Excision of Right Large Intestine, Via Natural or Artificial Opening Endoscopic |  | 0DBF8ZZ  0DBFFZZ |
| Excision of Right Large Intestine, Open Approach |  | 0DBF0ZZ |
| Excision of Right Large Intestine, Via Natural or Artificial Opening |  | 0DBF7ZZ |
| Excision of Left Large Intestine, Percutaneous Endoscopic Approach |  | 0DBG4ZZ |
| Excision of Left Large Intestine, Via Natural or Artificial Opening Endoscopic |  | 0DBG8ZZ |
| Excision of Left Large Intestine, Open Approach |  | 0DBG0ZZ |
| Excision of Left Large Intestine, Via Natural or Artificial Opening |  | 0DBG7ZZ |
| Excision of Cecum, Percutaneous Endoscopic Approach |  | 0DBH4ZZ |
| Excision of Cecum, Via Natural or Artificial Opening Endoscopic |  | 0DBH8ZZ  0DBHFZZ |
| Excision of Cecum, Open Approach |  | 0DBH0ZZ |
| Excision of Cecum, Via Natural or Artificial Opening |  | 0DBH7ZZ |
| Excision of Ascending Colon, Percutaneous Endoscopic Approach |  | 0DBK4ZZ |
| Excision of Ascending Colon, Via Natural or Artificial Opening Endoscopic |  | 0DBK8ZZ  0DBKFZZ |
| Excision of Ascending Colon, Open Approach |  | 0DBK0ZZ |
| Excision of Ascending Colon, Via Natural or Artificial Opening |  | 0DBK7ZZ |
| Excision of Transverse Colon, Percutaneous Endoscopic Approach |  | 0DBL4ZZ |
| Excision of Transverse Colon, Via Natural or Artificial Opening Endoscopic |  | 0DBL8ZZ |
| Excision of Transverse Colon, Open Approach |  | 0DBL0ZZ |
| Excision of Transverse Colon, Via Natural or Artificial Opening |  | 0DBL7ZZ |
| Excision of Descending Colon, Percutaneous Endoscopic Approach |  | 0DBM4ZZ |
| Excision of Descending Colon, Via Natural or Artificial Opening Endoscopic |  | 0DBM8ZZ |
| Excision of Descending Colon, Open Approach |  | 0DBM0ZZ |
| Excision of Descending Colon, Via Natural or Artificial Opening |  | 0DBM7ZZ |
| Excision of Sigmoid Colon, Via Natural or Artificial Opening |  | 0DBN7ZZ |
| Excision of Sigmoid Colon, Percutaneous Endoscopic Approach |  | 0DBN4ZZ |
| Excision of Sigmoid Colon, Via Natural or Artificial Opening Endoscopic |  | 0DBN8ZZ |
| Excision of Sigmoid Colon, Open Approach |  | 0DBN0ZZ |
| Excision of Sigmoid Colon, Via Natural or Artificial Opening |  | 0DBN7ZZ |
| Robotic Assisted Procedure of Trunk Region, Open Approach |  | 8E0W0CZ |
| Robotic Assisted Procedure of Trunk Region, Percutaneous Approach |  | 8E0W3CZ |
| Robotic Assisted Procedure of Trunk Region, Percutaneous Endoscopic Approach |  | 8E0W4CZ |
| Robotic Assisted Procedure of Trunk Region, Via Natural or Artificial Opening |  | 8E0W7CZ |
| Robotic Assisted Procedure of Trunk Region, Via Natural or Artificial Opening Endoscopic |  | 8E0W8CZ |
| Open robotic assisted procedure | 17.41 |  |
| Laparoscopic robotic assisted procedure | 17.42 |  |
| Percutaneous robotic assisted procedure | 17.43 |  |
| Endoscopic robotic assisted procedure | 17.44 |  |
| Thoracoscopic robotic assisted procedure | 17.45 |  |
| Other and unspecified robotic assisted procedure | 17.49 |  |
| Total Intra-Abdominal Colectomy | 45.8 |  |
| Laparoscopic total intra-abdominal colectomy | 45.81 | 0DTE4ZZ |
| Open total intra-abdominal colectomy | 45.82 | 0DTE0ZZ |
| Other and unspecified total intra-abdominal colectomy | 45.83 | 0DTE7ZZ, 0DTE8ZZ |
| Open And Other Partial Excision of Large Intestine | 45.7 |  |
| Open and other multiple segmental resections of large intestine | 45.71 | 0DBE0ZZ, 0DBE3ZZ, 0DBE7ZZ, 0DBE8ZZ |
| Open and other cecectomy | 45.72 | 0DTH0ZZ, 0DTH7ZZ, 0DTH8ZZ |
| Open and other right hemicolectomy | 45.73 | 0DTF0ZZ, 0DTF7ZZ, 0DTF8ZZ, 0DTK0ZZ |
| Open and other resection of transverse colon | 45.74 | 0DTL0ZZ, 0DTL7ZZ, 0DTL8ZZ, 0DTLFZZ |
| Open and other left hemicolectomy | 45.75 | 0DTG0ZZ, 0DTG7ZZ, 0DTG8ZZ, 0DTGFZZ |
| Open and other sigmoidectomy | 45.76 | 0DTN0ZZ, 0DTN7ZZ, 0DTN8ZZ, 0DTNFZZ |
| Other and unspecified partial excision of large intestine | 45.79 | 0DBE0ZZ, 0DBE3ZZ, 0DBE7ZZ, 0DBE8ZZ, 0DBGFZZ, 0DBLFZZ,  0DBMFZZ, 0DBNFZZ, 0DTMFZZ |

**Supplementary Table 2.** Risk Model for 30-day Mortality

| Variable | Estimate (SE) | Odds Ratio  (95% CI) | P-value |
| --- | --- | --- | --- |
| Intercept | -3.5471 (0.1696) | 0.00 (0.00-0.00) | <0.0001 |
| Age (years)  65-70  71-75  76-80  >80 (Reference) | -0.6138 (0.2042)  -0.4537 (0.1900)  -0.3683 (0.1966)  - | 0.541 (0.358-0.799)  0.635 (0.433-0.914  0.692 (0.465-1.007)  - | 0.0026  0.0170  0.0611  - |
| Urgent Surgery | 1.0959 (0.1484) | 2.992 (2.245-4.019) | <0.0001 |
| Comorbidities  CHF  CPD  Dementia  Liver disease  DM  Renal disease  Rheumatologic disease  Stroke | 0.8095 (0.1477)  0.1871 (0.1503)  0.2819 (0.2352)  0.6566 (0.2455)  -0.5206 (0.6195)  0.3977 (0.1542)  0.1293 (0.3626)  1.3631 (0.3852) | 2.247 (1.678-2.995)  1.206 (0.894-1.612)  1.326 (0.819-2.066)  1.928 (1.163-3.058)  0.594 (0.140-1.720)  1.488 (1.095-2.006)  1.138 (0.522-2.197)  3.908 (1.749-8.023 | <0.0001  0.2131  0.2307  0.0075  0.4007  0.0099  0.7213  0.0004 |
| Principal procedure  Open colectomy (Reference)  Laparoscopic colectomy  Excision via percutaneous approach  Resection via percutaneous approach  Excision via natural or artificial opening  Robotic surgery | -  -0.3503 (0.2444)  -0.8810 (0.4681)  -1.0369 (0.2407)  -0.0124 (0.1954)  -0.9329 (1.0184) | -  0.704 (0.425-1.113)  0.414 (0.144-0.938)  0.355 (0.215-0.556)  0.988 (0.665-1.433)  0.393 (0.022-1.842) | -  0.1519  0.0598  <0.0001  0.9494  0.3596 |

**Supplementary Table 3.** Risk Model for ICU Utilization

| Variable | Estimate (SE) | Odds Ratio  (95% CI) | P-value |
| --- | --- | --- | --- |
| Intercept | -1.3148 (0.0812) | 0.00 (0.00-0.00) | <0.0001 |
| Age (years)  65-70  71-75  76-80  >80 (Reference) | -0.2393 (0.0890)  -0.2130 (0.0868)  -0.0681 (0.0919)  - | 0.787 (0.661-0.937)  0.808 (0.681-0.958)  0.934 (0.780-1.118)  - | 0.0072  0.0141  0.4592  - |
| Sex  Male  Female (Reference) | 0.1376 (0.0651)  - | 1.147 (1.010-1.304)  - | 0.0378  - |
| Race  White (Reference)  Black  Hispanic  Other | -  0.2821 (0.1173)  0.2877 (0.2865)  -0.0115 (0.1585) | -  1.326 (1.052-1.666)  1.333 (0.750-2.318)  0.989 (0.720-1.342) | -  0.0162  0.3153  0.9422 |
| Transfer-in | 0.8540 (0.2916) | 2.349 (1.339-4.227) | 0.0034 |
| Urgent Surgery | 0.9030 (0.0671) | 2.467 (2.163-2.814) | <0.0001 |
| Comorbidities  CHF  CPD  Dementia  Liver disease  DM  Renal disease  Rheumatologic disease  Stroke | 0.7837 (0.0813)  0.3747 (0.0745)  0.3250 (0.1381)  0.3555 (0.1429)  0.9424 (0.3103)  0.0375 (0.0839)  0.1004 (0.1870)  0.7440 (0.3017) | 2.190 (1.867-2.568)  1.455 (1.256-1.683)  1.384 (1.055-1.813)  1.427 (1.076-1.885)  2.566 (1.409-4.788)  1.038 (0.880-1.223)  1.106 (0.762-1.588)  2.104 (1.166-3.827) | <0.0001  <0.0001  0.0186  0.0129  0.0024  0.6552  0.5912  0.0136 |
| Principal procedure  Open colectomy (Reference)  Laparoscopic colectomy  Excision via percutaneous approach  Resection via percutaneous approach  Excision via natural or artificial opening  Robotic surgery | -  -0.3784 (0.1111)  -0.5706 (0.1663)  -0.5602 (0.0867)  -0.1667 (0.1038)  0.5177 (0.2581) | -  0.685 (0.550-0.850)  0.565 (0.404-0.777)  0.571 (0.481-0.676)  0.846 (0.690-1.036)  1.678 (1.001-2.765) | -  0.0007  0.0006  <0.0001  0.1084  0.0448 |

**Supplementary Table 4.** Risk Model for Length of Stay

| Variable | Estimate (SE) | 95% CI | P-value |
| --- | --- | --- | --- |
| Intercept | 6.8163 (1.0423) | (4.7733-8.8592) | <0.0001 |
| Age (years)  65-70  71-75  76-80  >80 (Reference) | -0.5994 (0.1182)  -0.4800 (0.1161)  -0.2644 (0.1256)  - | (-0.3678 - -0.8310)  (-0.2526 - -0.7075)  (-0.0183 - -0.5105)  - | <0.0001  <0.0001  0.0353  - |
| Race  White (Reference)  Black  Hispanic  Other | -  0.4832 (0.1636)  0.7187 (0.4112)  -0.1440 (0.2099) | -  (0.1626-0.8039)  (0.0872-1.5245)  (-0.5554-0.2673) | -  0.0031  0.0805  0.4925 |
| Urgent Surgery | 3.7215 (0.0949) | (3.5355-3.9074) | <0.0001 |
| Comorbidities  CHF  CPD  Dementia  Liver disease  DM  Renal disease  Rheumatologic disease  Stroke | 1.0748 (0.1191)  0.5659 (0.1050)  0.8352 (0.2018)  -0.0435 (0.2050)  -0.0123 (0.4411)  0.6639 (0.1162)  0.5415 (0.2566)  -0.1465 (0.4410) | (0.8414-1.3081)  (0.3601-0.7717)  (0.4398-1.2307)  (-0.4452-0.3583)  (-0.8769-0.8524)  (0.4361-0.8918)  (0.0386-1.0444)  (-1.0108-0.7179) | <0.0001  <0.0001  <0.0001  0.8321  0.9778  <0.0001  0.0348  0.7398 |
| Principal procedure  Open colectomy (Reference)  Laparoscopic colectomy  Excision via percutaneous approach  Resection via percutaneous approach  Excision via natural or artificial opening  Robotic surgery | -  -0.6309 (0.1446)  -1.2648 (0.2007)  -1.4734 (0.1104)  -0.8659 (0.1462)  -1.1540 (0.3781) | -  (-0.3476 - -0.9143)  (-0.8714 - -1.6581)  (-1.2571 - -1.6897)  (-0.5794 - -1.1523)  (-0.4129 - -1.8951) | -  <0.0001  <0.0001  <0.0001  <0.0001  0.0023 |

**Supplementary Table 5.** Risk Model for Cost of Care

| Variable | Estimate (SE) | 95% CI | P-value |
| --- | --- | --- | --- |
| Intercept | 76824.62 (17032.93) | (43440.70-110208.5) | <0.0001 |
| Age (years)  65-70  71-75  76-80  >80 (Reference) | 1427.48 (1123.065)  2421.89 (1100.246)  2720.83 (1189.574)  - | (-773.688-3628.65)  (265.449-4578.33)  (389.312-5052.36)  - | 0.2037  0.0277  0.0222  - |
| Sex  Male  Female (Reference) | 2346.36 (828.2821)  - | (722.956-3969.76)  - | 0.0046  - |
| Race  White (Reference)  Black  Hispanic  Other | -  4143.03 (1550.363)  154.850 (3893.886)  -4138.218 (1988.817) | -  (1104.38-7181.69)  (-7477.027-7786.73)  (-240.2093 - -8036.227) | -  0.0075  0.9683  0.0375 |
| Transfer-in | 13494.3 (4045.321) | (5565.66-21423.0) | 0.0009 |
| Comorbidities  CHF  CPD  Dementia  Liver disease  DM  Renal disease  Rheumatologic disease  Stroke | 10578.5 (1128.183)  4311.64 (994.5190)  -2054.775 (1910.934)  3823.95 (1941.212)  13413.2 (4177.929)  2363.38 (1106.271)  4675.60 (2437.898)  -919.2871 (4180.307) | (8367.34-12789.7)  (2362.41-6260.86)  (-5800.136-1690.59)  (19.2493-7628.66)  (5224.66-21601.8)  (195.130-4531.63)  (-102.5961-9453.79)  (-9112.538-7273.96) | <0.0001  <0.0001  0.2823  0.0489  0.0013  0.0327  0.0551  0.8259 |
| Principal procedure  Open colectomy  Laparoscopic colectomy  Excision via percutaneous approach  Resection via percutaneous approach  Excision via natural or artificial opening  Robotic surgery (Reference) | -3266.705 (3582.646)  -3648.929 (3732.752)  -9359.987 (3946.453)  -9974.697 (3619.575)  -4750.417 (3751.720)  - | (-10288.56-3755.15)  (-10964.99-3667.13)  (-1625.082 - -17094.89)  (-2880.460 - -17068.93)  (-12103.65-2602.82)  - | 0.3619  0.3283  0.0177  0.0059  0.2054  - |

**Supplementary Table 6.** Regression Analysis after adjusting for patient covariates

| *Outcome* | OR (95% CI) | *P* value |
| --- | --- | --- |
| 30-day mortality  Unranked hospital  Ranked hospital | Ref.  OR: 0.55 (0.47-0.64) | <0.001 |
| *Outcome* | Difference (95% CI) | *P* value |
| Difference in mean adjusted cost of care ($) | 10,352 (5,410-15,294) | <0.001 |
